# The effect of physical exercise on the spiritual well-being of older adults-mediating role of self-care ability

**DOI:** 10.1371/journal.pone.0342762

**Published:** 2026-02-12

**Authors:** Meng Wang, Xiaolei Yang, Yanhong Su

**Affiliations:** Key Laboratory of Sports Human Science in Liaoning Province, College of Physical Education, Liaoning Normal University, Dalian, China; School of Nursing Sao Joao de Deus, Evora University, PORTUGAL

## Abstract

**Purpose:**

With the increasing aging of the population, older adults over the age of 65 constitute the largest and most rapidly expanding population group in the world, and the quality of survival of older adults is generally decreasing. A growing number of researchers have shown that physical exercise is positively associated with spiritual well-being and self-care ability in older adults, thereby affecting their health status and quality of life. The purpose of this study was to investigate the effect of physical exercise on the spiritual well-being of older adults and the mediating role of self-care ability.

**Patients and methods:**

The research data collection period in this study was from June 8, 2024, to Sept 2024. We surveyed 405 urban older adults and used Pearson correlation coefficients to analyze the relationship between physical exercise, spiritual well-being, and self-care ability. Mediation analysis was used to explore the mediating role of self-care ability in physical exercise and spiritual well-being.

**Results:**

Physical exercise is positively related to the spiritual well-being index and is related to the mediating effect of self-care ability.

**Conclusion:**

This study shows that physical exercise is positively correlated with both spiritual well-being and self-care ability of the elderly, and affects the spiritual well-being index through the mediating effect of self-care ability. Physical exercise is a proactive behavior, which is conducive to enhancing the physical and spiritual health and well-being of the elderly under the advocacy of “active aging”.

## Introduction

Countries around the world are experiencing demographic changes as their populations age, with the total number of people aged 65 and older reaching 703 million in 2019 and increasing to 1.5 billion in 2050 [1,2]. The seventh national census shows that China’s aging population reached 264 million in 2020, accounting for 18.7% of the country’s total population [3,4]. With the deepening of China’s population aging and the implementation of the Health China strategy, the issue of aging health has become a hot issue of general concern to society. Therefore, active aging has become an important initiative to alleviate the aging problem. Both the elderly in the community and the elderly in institutions are commonly faced with physical decline, mental illness, economic loss, and social exclusion, which make the quality of life of the elderly significantly lower and thus lead to some spiritual health problems [5]. Studies show that older adults are prone to negative emotions such as loneliness, depression, and even suicide [5]. A systematic review and meta-analysis in 2022 showed that the global prevalence of depression in older adults was 28.4% [6], and accounted for 36% of all suicides in China [7]. More than 100,000 older adults die by suicide each year, and most are experiencing depression [8], decreased quality of life due to physical weakness or illness, in addition to depression [9]. Therefore, the spiritual health of the elderly needs urgent attention.

Spiritual well-being is an important predictor of mental health in older adults and has become an important indicator of a person’s positive health outcomes and perceived quality of life [10]. This study focuses on spiritual well-being as the primary outcome variable. It is essential to distinguish spiritual well-being from the broader concept of mental health. Mental health encompasses the overall psychological state, including the absence of mental illness and the presence of positive psychological functioning. In contrast, spiritual well-being of the elderly refers to the pleasurable spiritual experience of meaning (value) and hope (strength) that individuals experience from within when dealing with their relationship with others, society, and the environment, etc [11]. Spiritual well-being is different from general subjective well-being and psychological well-being, emphasizing the individual’s experience of well-being at a higher level, including the individual’s physical health [12], and mental health [13], especially important for successful aging of the elderly [14].

Increasingly, researchers are recognizing that improving the spiritual well-being of older adults can help improve their health status and quality of life. Physical exercise has emerged as a key intervention to promote spiritual health in aging populations. Physical exercise prevents chronic diseases, strengthens bones and muscles, helps maintain a healthy weight, and enhances overall well-being. Although there is growing evidence that physical exercise is associated with improved spiritual health outcomes in older adults, the specific mechanisms through which physical exercise affects spiritual health remain unclear.

Self-care ability is defined in Orem’s self-care theory as the ability of an individual to engage in self-care activities or self-care. The selection of self-care ability as an intermediate variable is based on the following theoretical considerations: (1) According to stress and coping theory, the health challenges faced by the elderly require mitigation through the enhancement of personal coping resources, and self-care ability is an important coping resource [15]; (2) Self-efficacy theory suggests that an individual’s belief in their own capabilities influences their behavioral choices and emotional state, and self-care ability reflects older adults’ confidence in managing their own health [16]; (3) Activity theory posits that older adults maintain quality of life and spiritual health by engaging in active participation and self-management [17]. The purpose of this theory is to maximize and promote patients’ self-care abilities around nursing goals, thereby accelerating their recovery and improving their quality of life.

Physical education enhances self-care in a few ways: it improves physical function and mobility, increases confidence to perform daily activities, develops problem-solving skills, and promotes autonomous health management. Although previous research has established that physical exercise is beneficial for spiritual health and self-care ability is important for healthy aging, the mediating pathways linking these variables have not been systematically investigated. Based on self-care theory and existing empirical evidence, this study focuses on the mediating role of self-care ability, providing a new perspective for understanding the underlying mechanisms through which physical exercise promotes spiritual health in older adults.

## Literature review and hypothesis development

### Basic theoretical framework

This study constructs a theoretical framework linking physical exercise, self-care ability, and spiritual well-being based on the Successful Aging Theory and Self-Determination Theory. The Successful Aging Theory emphasizes that physical health, cognitive function, and active participation in life are core elements of well-being in older adults [18,19]. This theory posits that older adults can maintain a high quality of life and spiritual health during the aging process by maintaining an active lifestyle and self-management abilities. Self-Determination Theory highlights that an individual’s autonomy, competence, and relatedness form the foundation of psychological well-being [20]. Physical exercise, as an active health-promoting behavior, not only directly enhances physical health but also is associated with spiritual well-being by strengthening an individual’s self-care ability. (reflecting autonomy and competence).

### Physical exercise and spiritual well-being

Spiritual well-being is evaluated in terms of two dimensions: self-efficacy as well as meaningfulness of life for older adults and those with chronic illnesses. Self-efficacy is the individual’s belief in his or her ability to perform the behaviors necessary to produce specific performance achievements [21]. People with a high sense of self-efficacy can have a more positive life course [22]. An active lifestyle may have a positive impact on an individual. Among healthy older adults, individuals with high self-efficacy are more likely to benefit from health education programs than those with low self-efficacy [23]. It shows that spiritual well-being plays a key role in guiding the lives of older adults, helping them to clarify the meaning of life and cope with negative circumstances, supporting them spiritually and helping to generate positive emotions to cope with the distress caused by aging, and having a positive impact on the quality of life and mortality outcomes of older adults [24,25].

The elderly are prone to losing their ability to live independently due to spiritual or physical problems, and suffer from isolation, loss of independence, loneliness, and other psychological distress due to retirement or disability, and other reasons that lead to a decline in socio-economic status [26], more likely to develop psychological problems such as depression and anxiety. Depression and death anxiety in older adults have an impact on their level of spiritual well-being. Depression is negatively associated with spiritual well-being [27]. Physical exercise, as a way for people to proactively prevent disease and improve their health, has a positive impact in promoting physical and spiritual health, quality of life, and well-being, as well as reducing depression in older adults.

The more the elderly participate in physical exercise, the higher their spiritual well-being. Some studies point out that physical exercise is a protective factor that promotes the development of individual psychological resilience, and physical exercise first directly buffers the sense of disuse brought about by the decline in physical function and helps individuals to enhance their sense of self-control and self-efficacy [28]. In addition, it can help older adults reduce risk factors and increase an individual’s sense of control and value [28]. Chang et al. also found that physical exercise had a positive effect on improving the spiritual well-being of older adults [29]. In addition, when the frequency of physical exercise is high, the incidence of cognitive decline in older adults is reduced [30]. Therefore, older adults can also participate in physical exercise to delay the decline of physical and cognitive function, to promote spiritual well-being, which in turn improves the health status and quality of life of older adults. In the context of the growing aging problem, it is imperative to explore the relationship between physical exercise and spiritual well-being in the elderly and its underlying mechanisms of action. Therefore, we propose the following hypothesis:

**H1.** *Physical exercise will be positively associated with spiritual well-being in older adults.*

### Role of self-care ability

Self-care ability is defined in Orem’s self-care theory as the ability of a person to perform self-care activities or self-care [31].Studies have shown that the level of self-care ability of elderly patients with chronic diseases may be related to patients’ labor capacity, with patients who have some physical labor having relatively high self-care ability and those who generally have insufficient labor capacity having poor self-care ability. Under the influence of health behaviors, enhancing the self-care ability of the elderly also improves their ability to self-manage chronic diseases, reduces the need for external resources, and facilitates the rational allocation of resources [32]. There is a positive correlation between self-care ability and health behaviors. Patients with high levels of self-care ability are usually better able to perform health behaviors, and conversely, good performance of health behaviors can effectively contribute to the development of self-care ability [33–35].

Physical exercise, as one of the healthy behaviors, actively enhances the daily self-care ability of the elderly, is good at self-behavior management, and focuses on physical and spiritual health, thus slowing down disease progression [36,37]. Therefore, we propose Hypothesis 2:

**H2.** *Physical exercise will be positively associated with self-care ability in older adults.*

The level of self-care ability can be influenced by a variety of factors, including innate influences and the influence of acquired learning and training, including specific factors such as psychological quality, physical health, cultural background, level of education, social life environment, and public service resources [38]. Older adults describe self-sufficiency as the ability to perform activities of daily living, to take care of one’s health, and to work independently (active living). Having the ability to control one’s own health means functioning well physically, staying healthy through self-management despite physical frailty, and maintaining good spiritual health in the context of such life-changing situations. Older adults value their ability to live their own lives without becoming a burden to their families, and to enhance their well-being and quality of life [39].

Previous studies have also shown that the onset of chronic diseases in the elderly affects their physical exercise functions and their ability to take care of themselves, which also affects their spiritual well-being and reduces their quality of life [40]. In addition, self-care in heart failure patients had a significant effect on the relationship between depression and quality of life, with quality of life depending on the level of heart failure symptoms and the degree of self-care involvement; the better the self-care, the less the effect of physical symptoms and depression on quality of life [41,42]. It suggests that the decline in self-care may be closely related to the physical and spiritual well-being of older adults.

Physical exercise promotes the physical and spiritual health of the elderly, which is important for improving their well-being of the elderly and achieving the purpose of improving their quality of life [30]. Therefore, self-care ability may be an intrinsic mechanism of action of physical exercise to enhance the spiritual well-being of older adults. Therefore, we propose Hypothesis 3:

**H3.** *Self-care ability positively mediates physical exercise and spiritual well-being in older adults.*

**Fig 1:**
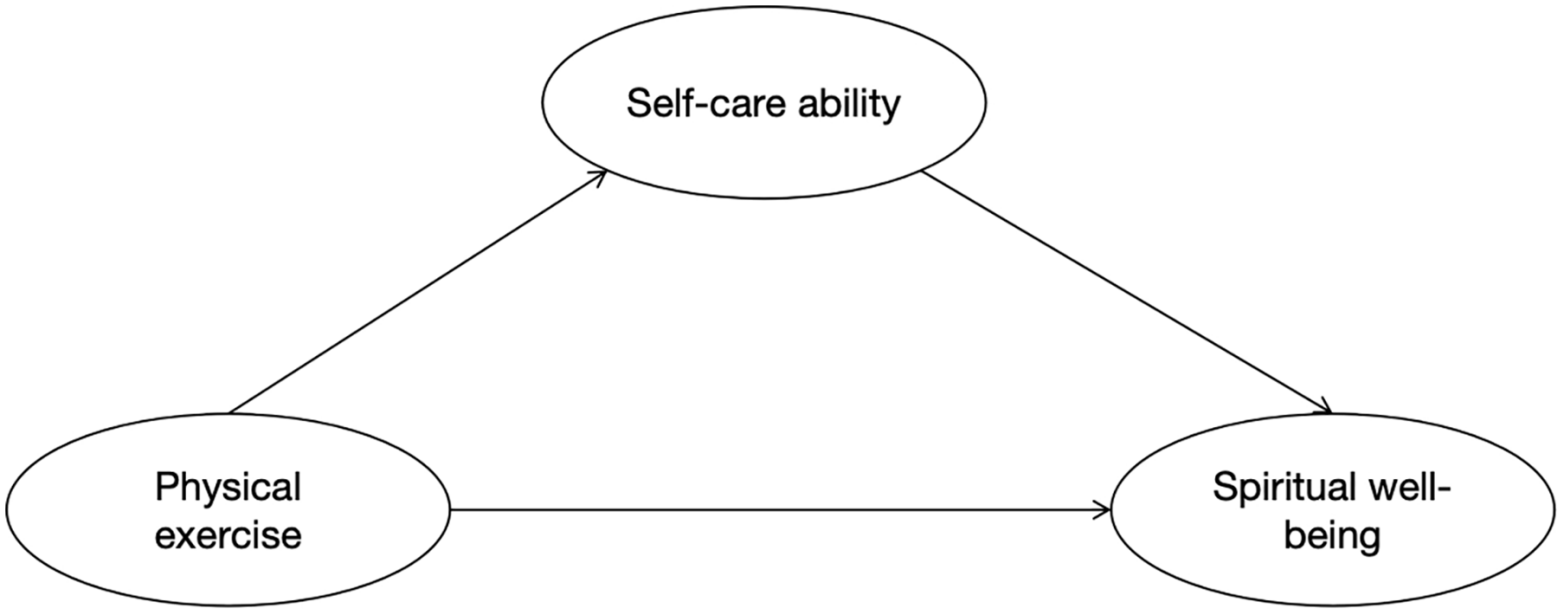
Diagram of the intermediary role model.

The research model is presented in Fig 1.

## Materials and methods

### Research subjects

The study sample was selected from urban older adults aged 65–80 years in Liaoning Province, China, for the survey. Given the feasibility of obtaining the complete list of the entire province, we adopted a multi-stage random sampling based on geographical regions to approximate this framework. Specific sampling process: First stage (Selecting cities): From all prefecture-level cities in Liaoning Province, three cities (Shenyang, Dalian, and Anshan) were randomly selected using the simple random sampling method. Phase Two (Community Selection): In each selected city, from the list of all urban communities, the simple random sampling method is used again to randomly select 4 communities from each, totaling 12 communities. Phase Three (Selecting Respondents): In each selected community, elderly people who meet the inclusion and exclusion criteria are randomly invited to participate in the survey from the list of members of the community’s elderly activity center, residents’ health records, or the list of permanent elderly residents provided by community staff.

### Inclusion criteria

(1) Be between 65 and 80 years old;
(2) The permanent residence time in the surveyed community is ≥ 1 year;
(3) Clear consciousness, possessing basic communication and comprehension skills, and capable of completing questionnaires independently or with the assistance of investigators;
(4) Informed consent and voluntary participation in this study.

### Exclusion criteria

(1) Suffering from severe cognitive impairment (such as Alzheimer’s disease confirmed by family members or community doctors, moderate or severe dementia), or having a serious mental illness and being unable to cooperate in completing the questionnaire survey;
(2) Suffering from serious physical diseases (such as advanced cancer, severe heart failure, recent myocardial infarction or stroke, etc.), and their physical condition does not allow them to engage in any form of physical exercise;
(3) There are severe audio-visual impairments, making it impossible to communicate effectively with the investigator;
(4) Those who have been bedridden for a long time or have extreme difficulty moving around, and are completely unable to take care of themselves in daily life.

A random sample of respondents was used in this study, which was approved by the Ethics Committee of Liaoning Normal University (LL2024090). The study complied with the Declaration of Helsinki. Participation in this study was voluntary and conducted anonymously. Therefore, respondents who chose “agree” when they gave informed consent were considered to have consented to participate in our survey. The research data collection period in this study was from June 8, 2024, to Sept 2024. During the survey, 500 questionnaires were distributed, and 405 valid questionnaires were returned, with a valid return rate of 88% (Table 1).

**Table 1:** The demographic breakdown of categorical variables (N = 405).

Variables		N	%
Gender	Male	176	43.50
	Female	229	56.50
Age	65–70	137	33.83
	71–75	122	30.12
	76–80	146	36.05

**Note:** As can be seen from the above table, among the sample of this survey, 176 (43.5%) were male and 229 (56.5%) were female; in terms of age distribution, 137 (33.83%) were aged 65–70, 122 (30.12%) were aged 71–75, and 146 (36.05%) were aged 76–80.

### Research tools

Physical Exercise Rating Scale (PARS-3). The scale was developed by the Japanese psychologist Koyo Hashimoto [43]. The Physical Exercise Rating Scale, revised by Liang Deqing et al. of Wuhan Institute of Physical Education, investigated the participation of the subjects in physical exercise in this study [44]. The scale examines the amount of physical exercise of the subjects in terms of intensity, duration, and frequency of exercise, and measures their level of physical exercise participation by the amount of physical exercise. The study used a 5-point Likert scale (1 –5), and the lower the subject’s score, the lower the physical exercise level. In this study, the reliability test was conducted, and the results showed that the Cronbach’s alpha coefficient was 0.904, and the model fit results of the validity test were RMSEA = 0.052, IFI = 0.93, TLI = 0.91, CFI = 0.90, and GFI = 0.91, which indicated that the reliability of the scale was good (Table 2).

**Table 2:** Discriminant validity of each dimension of physical exercise level.

	Exercise intensity	Exercise time	Exercise frequency
Exercise intensity	0.862a		
Exercise time	.442***	0.854a	
Exercise frequency	.351***	0.424***	0.846a

**Note:** *p < 0.05, **p < 0.01, ***p < 0.001; the diagonal is the square root of AVE.

From the above table, it can be seen that the convergent validity (AVE) of the three dimensions of physical exercise intensity, exercise time and exercise frequency in the physical exercise rating scale are 0.743, 0.729 and 0.715, respectively, and there is a significant correlation among the dimensions, but the absolute value of the correlation coefficient is less than 0.5 and less than the square root of the corresponding AVE, which means that the three dimensions have a certain correlation, but also have a certain degree of differentiation between each other, indicating that the scale data are ideally differentiated.

Self-Care Ability Assessment Scale (ASAS-R-C): is the scale was developed by Swedish scholar Soderhamn O in 1996 [31], Chinese translation by Lina Guo et al. demonstrated good reliability and validity in Chinese population [45], the scale has 3 dimensions, general self-care, developmental self-care, and self-care in ill health, with 15 entries, 4 of which (4th, 11th, 14th, and 15th) are reverse scored using a Likert scale of 1–5, with higher scores indicating the respondent’s greater self-care ability. The overall Cronbach’s alpha coefficient of the scale in this study was 0.883. The results of the follow-up validation factor analysis showed that the RMSEA = 0.057, IFI = 0.923, TLI = 0.912, CFI = 0.916, GFI = 0.904, the number of indicators was within the standard acceptable range, and the structure of the questionnaire validity is good (Table 3).

**Table 3:** Discriminant validity of self-care competency dimensions.

	General self-care	Developmental self-care	Self-care in poor health
General self-care	0.819a		
Developmental self-care	.367***	0.826a	
Self-care in poor health	.441***	.375***	0.805a

**Note:** *p < 0.05, **p < 0.01, ***p < 0.001; the diagonal is the square root of AVE.

From the above table, it can be seen that the convergent validity (AVE) of the three dimensions of self-care ability questionnaire, general self-care ability, developmental self-care ability and self-care ability in poor health, are 0.671, 0.682 and 0.648 respectively and there is a significant correlation between the dimensions, but the absolute value of the correlation coefficient is less than 0.5 and less than the corresponding AVE square root of the corresponding AVE, which means that the three dimensions have a certain correlation, but have a certain degree of differentiation among each other, indicating that the differentiation of the scale data is ideal.

The Spirituality Index of Well-Being Scale (SIWB) was developed by Daaleman et al. in 2002 to assess the health-related quality of life of chronically ill and elderly people based on the definition of spiritual well-being [46]. The scale consists of 2 dimensions: self-efficacy, which measures an individual’s functional life self-efficacy, and meaning of life, which assesses a person’s perception of the meaning of his or her life. There are 12 items in total, with each dimension containing 6 items each. A 5-point Likert scale was used to rate the scores from “strongly disagree=1” to “strongly agree=5”, with higher scores indicating greater spiritual well-being. The Cronbach’s alpha coefficients for the two dimensions and the total scale were above 0.8. The results of the follow-up validation factor analysis showed that the RMSEA = 0.041, IFI = 0.903, TLI = 0.935, CFI = 0.909, and GFI = 0.917, the number of each indicator was within the standard acceptable range, and the questionnaire had good construct validity (Table 4).

**Table 4:** Discriminant validity of spiritual well-being index.

	Self-efficacy	Meaning of Life
Self-efficacy	0.867a	
Meaning of Life	.432***	0.882a

**Note:** *p < 0.05, **p < 0.01, ***p < 0.001; the diagonal is the square root of AVE.

From the above table, it can be seen that the convergent validity (AVE) of the two dimensions of self-efficacy and meaning of life in the spiritual well-being index questionnaire are 0.751 and 0.778 respectively and there is a significant correlation between the dimensions, but the absolute value of the correlation coefficient is less than 0.5 and less than the square root of the corresponding AVE, which means that there is a certain correlation between the two dimensions, but have a certain degree of differentiation between each other, indicating that the scale data are ideally differentiated.

The data obtained from the survey were all analyzed using SPSS 23.0, descriptive statistics showed the statistical characteristics of the participants, and correlation and mediating effect analyses showed how physical exercise level affected self-care ability and spiritual well-being index, and the mediating role played by self-care ability in this.

### Control and testing of common method deviations

Based on procedural controls for possible common method bias (e.g., anonymous completion), validated factor analysis was further conducted using AMOS 23.0, with the questions used in the Physical exercise Rating Scale and the Self-Care Competence Scale as exogenous variables for factor analysis, resulting in the following fit indices: x2/df = 14.7, RMSEA = 0.243, NFI = 0.315, GFI = 0.342, CFI = 0.445, and IFI = 0.304, indicating that a single common factor does not adequately account for the covariance among items. However, we admit that this test has limitations and it is impossible to determine the exclusion of common methodological biases. Therefore, when interpreting the results of this study, the variance of the common method should be considered as a potential limitation and verified through future utilization of multiple data sources or longitudinal designs.

### Data analyses

The data were analyzed using SPSS 23.0 and the macro program PROCESS v3.4.1. Descriptive statistics and correlation analysis were conducted using SPSS 23.0, and PROCESS v3.4.1 was used to test mediators. Bootstrap sampling was repeated 5,000 times, with the confidence interval set at 95%. The Pearson correlation coefficient was used to test the relationship between physical exercise and spiritual well-being. Before this, the Shapiro-Wilk test and visual inspection of Q-Q plots were used to confirm the normality of the key continuous variable (PARS-3, ASAS-R-C and SIWB scores) data. Since all variables conform to the normality hypothesis (Shapiro-Wilk test, *p* > 0.05), calculate the Pearson’s correlation coefficient. Calculate the variance inflation factor (VIF) for all predictor variables in the regression model to test for multicollinearity. All VIF values were below 2.0, indicating that multicollinearity was not a concern. Demographic variables (including gender and age) were included as control variables in the analysis.

## Results

### Descriptive statistics and correlation analysis among variables

Correlation analysis was used to analyze the relationship between physical exercise level, self-care ability and spiritual well-being index, and the results showed that there was a significant positive correlation between physical exercise level, self-care ability and spiritual well-being index. (Table 5) Among them, the level of physical exercise was positively correlated with the self-care ability in life (r = 0.625, *p* < 0.001) and positively correlated with the index of spiritual well-being (r = 0.605, *p* < 0.001). Self-care ability is also positively correlated with the spiritual well-being index (r = 0.585, *p* < 0.001). The data confirm that all dimensions show a significant positive correlation, but the relationship among the three dimensions needs further testing to determine the mediating effect and further reveal its internal influence mechanism.

**Table 5:** Results of correlation analysis of physical exercise level, self-care ability and spiritual well-being index.

	M	SD	1	2	3
1 Physical exercise level	3.283	0.805	1		
2 Self-care ability	3.283	0.768	.625***	1	
3 Spiritual well-being index	3.187	0.877	.605***	.585***	1

**Note:** *p < 0.05, **p < 0.01, ***p < 0.001.

### Intermediation effect test

First, Model 4 in the SPSS macro prepared by Hayes was used (Model 4 is a simple mediated model) [47,48], the mediating effect of self-care ability in the relationship between physical exercise level and spiritual well-being index was examined, controlling for Gender, Age. These variables were included as covariates because prior research suggests they are potential confounders. By controlling for these factors, we aimed to isolate the specific relationship between our variables of primary interest. The results showed (Table 6) that the positive correlation effect of physical exercise level on spiritual well-being index was significant (β = 0.668, t = 14.769, *p* < 0.001), and the direct positive correlation effect of physical exercise level on spiritual well-being index remained significant when mediating variables were put in (β = 0.435, t = 7.978, *p* < 0.001). The positive correlation effect of physical exercise level on self-care ability was significant (β = 0.621, t = 15.796, *p* < 0.001), as was the positive correlation effect of self-care ability on spiritual well-being index (β = 0.375, t = 6.900, *p* < 0.001).

**Table 6:** Regression analysis of the relationship between variables in the intermediary model.

Variables	Spiritual well-being index		Spiritual well-being index		Self-care ability	
	β	t	β	t	β	t
Gender	0.170	2.255*	0.228	2.888**	0.156	2.268*
Age	−0.061	−1.352	−0.041	−0.860	0.054	1.296
Physical exercise level	0.435	7.978***	0.668	14.769***	0.621	15.796***
Self-care ability	0.375	6.900***				
R²	0.454		0.389		0.398	
F	83.111***		85.056***		88.307***	

**Note:** *p < 0.05, **p < 0.01, ***p < 0.001.

**Fig 2:**
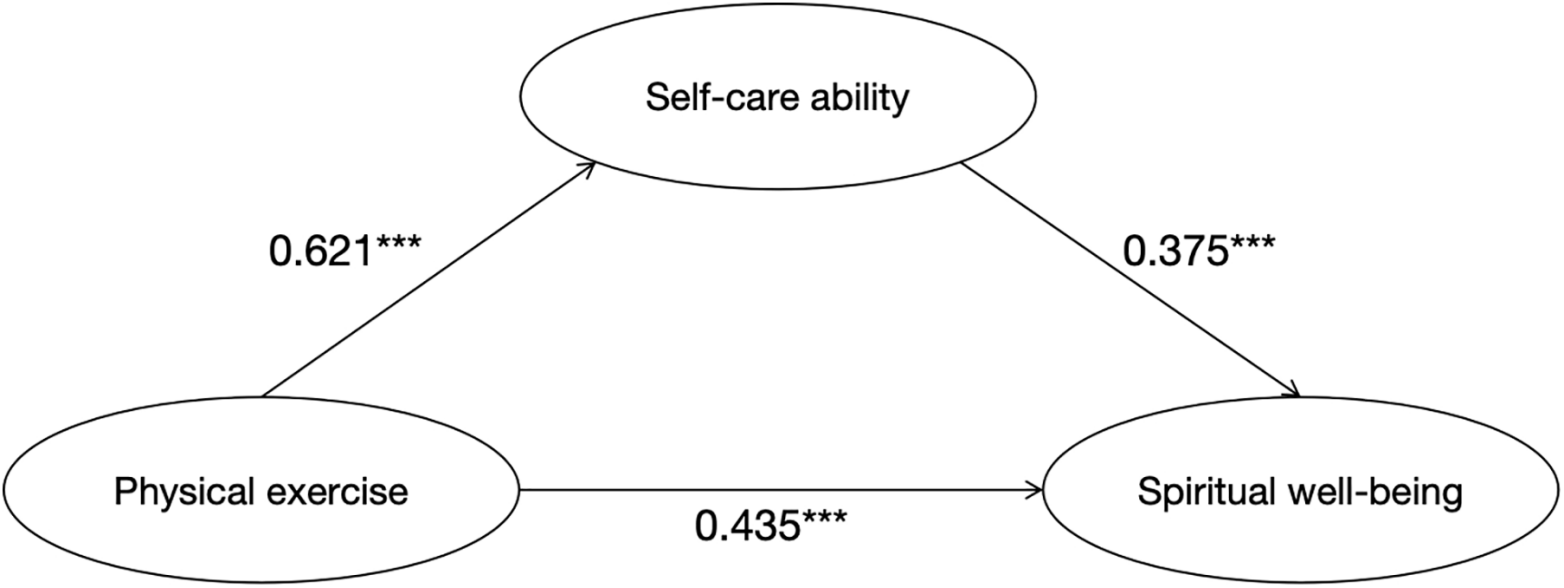
Mediation model of physical exercise level and spiritual well-being index.

In this study, the bias-corrected nonparametric percentage Bootstrap test was used to calculate 95% confidence intervals for specific mediating effects, comparison mediating effects, and total mediating effects after 5000 replicate samples [49], the upper and lower limits of the bootstrap 95% confidence interval for the direct effect of physical exercise level on the spiritual well-being index and the mediating effect of self-care ability did not contain 0, indicating that physical exercise level not only associated with the spiritual well-being index directly, but also plays a mediating role in self-care ability. This direct effect (0.435) and mediating effect (0.233) accounted for 65.12% and 34.88% of the total effect (0.668), respectively. The specific paths of the effect of physical exercise level on the spiritual well-being index are shown in Table 7 and Fig 2.

**Table 7:** Mediating effects of physical exercise level, self-care ability, and spiritual well-being index.

	Effect	BootSE	BootLLCI	BootULCI	Relative Effect Value
TOTAL	0.668	0.045	0.579	0.757	
Direct Effect	0.435	0.055	0.328	0.542	65.12%
Indirect Effect	0.233	0.046	0.147	0.330	34.88%

Note: *p < 0.05, **p < 0.01, ***p < 0.001.

## Discussion

The present study employed mediation analysis to investigate the complex relationships among physical exercise, self-care ability, and spiritual well-being in older adults. The findings confirm our central hypothesis that self-care ability serves as a significant mediator between physical exercise and spiritual well-being. This theoretical framework provides a more nuanced understanding of how physical exercise intervention affects the spiritual health of the elderly. The results of Post-hoc Power Analysis indicated that under the sample size (N = 405) and the observed effect size of this study, the statistical Power exceeded 0.99, proving that this study has sufficient ability to detect the real effect.

Health promotion behaviors are health management activities for healthier living that stimulate people’s health potential and promote their physical and spiritual well-being health [50]. Physical exercise as part of health-promoting behavior may be influenced by family members’ care, personal attitudes toward health, and physical exercise [51]. Older adults’ spiritual well-being contains two dimensions (self-efficacy and meaningfulness of life), and according to Lee and Oh, confidence in performing health behaviors, i.e., self-efficacy, plays a critical role in influencing decisions about health-promoting behaviors [52]. And self-efficacy and physical exercise behavior are mutually beneficial [53], that is, self-efficacy maintains motor intentions and behaviors, and regular exercise in turn helps to improve self-efficacy. People with high self-efficacy prefer healthy lifestyle behaviors. And healthy life behaviors (physical exercise) may further increase self-efficacy and improve quality of life and psychological disorders, forming a virtuous circle. Self-efficacy as a part of spiritual well-being suggests that physical exercise may serve as a good intervention for spiritual health in older adults.

Our results establish a significant positive relationship between physical exercise and spiritual well-being in older adults. This direct effect can be interpreted through multiple theoretical lenses. Primarily, physical exercise appears to function as a protective factor that enhances psychological resilience. As demonstrated by YANG et al., exercise directly counters the sense of disuse stemming from age-related physical decline while simultaneously strengthening self-control and self-efficacy. This dual mechanism aligns well with the conceptualization of spiritual well-being as comprising both self-efficacy and meaningfulness of life [28]. In addition, it can help older adults reduce risk factors and increase an individual’s sense of control and value [28]. The literature consistently supports this relationship. Intervention studies by Cheung et al. (2016) confirmed that structured physical exercise programs significantly improve both dimensions of spiritual well-being [54]. Further evidence suggests that exercise-induced improvements in spiritual well-being may underlie broader benefits, including reduced depression and anxiety [55,56], extended longevity [57], and enhanced social connectivity [58,59]. Importantly, the correlation between spiritual well-being and physical functioning suggests a bidirectional relationship [60], while its connection to quality of life [61] indicates that spiritual well-being may serve as a crucial mechanism through which physical exercise enhances overall life satisfaction in later years.

Beyond this direct relationship, our mediation analysis reveals the crucial role of self-care ability. Self-care ability, defined as the natural decision-making processes that affect individuals in maintaining physiological stability (referring to self-care maintenance behaviors such as healthy lifestyle, adherence to treatment regimens, and monitoring of symptoms) and managing symptoms (referring to self-care management behaviors such as identifying health changes, implementing treatment strategies, and evaluating implemented treatments), self-care ability represents a critical component of chronic disease management and overall health maintenance in aging populations [38].

Self-care ability is closely related to negative emotions and quality of life in the elderly. Some elderly patients with chronic diseases believe that their lack of self-care ability is a drag on their families and the country, and therefore show signs of resistance to treatment, so improving the self-care ability of the elderly and patients with chronic diseases is necessary to improve treatment compliance and enhance their sense of well-being [62,63]. Previous studies have shown that 6 months of aerobic exercise significantly improved the self-care ability and quality of life of elderly patients with chronic diseases. Our research results show that physical exercise is significantly positively correlated with the self-care ability of the elderly. This relationship may be explained in several ways. First, exercise can directly enhance physical ability and make one more independent in daily activities. Secondly, as indicated by the Aminuddin et al Institute, physical exercise enhances patients’ participation in self-care and improves treatment compliance [36,64]. This indicates that exercise not only enhances physical capabilities but also cultivates proactive management of spiritual health.

The connection between self-care ability and spiritual well-being is the final link in our theoretical model. Studies show that the improvement of self-care ability is associated with the enhancement of self-efficacy [65], the reduction of negative emotions [66], and the improvement of quality of life and happiness [66]. Our research findings expand this understanding, demonstrating that self-care ability influences spiritual well-being through two dimensions: establishing self-efficacy through successful health management and enhancing the meaning of life by maintaining autonomy and goals.

In conclusion, the mediating effect analysis in this study provides a theoretical framework for understanding the impact of physical exercise on the spiritual well-being of the elderly. The results of the mediated effects analysis in this study showed that physical exercise not only is positively correlated with the spiritual well-being of older adults, can also influence their spiritual well-being through self-care ability. First, older adults with higher physical exercise had better health behavior levels and thus perceived more self-efficacy and self-worth. Physical exercise and self-care ability were positively correlated. Only when older adults themselves perceive improved self-care ability can they better buffer stress, such as reducing negative emotions like depression and anxiety, and improve positive emotions to enhance the quality of survival and interpersonal relationships of older adults, thus improving spiritual well-being and overall quality of life in general.

## Implications

This study enriches and expands the application area of the theoretical model of physical exercise and health behavior promotion in the elderly. The decline of physical exercise in the elderly is accompanied by the decline of quality of life and depression in the elderly, and the spiritual health of the elderly has become an urgent problem. The mechanism of action of physical exercise as one of the means of spiritual well-being enhancement is yet to be studied. Although self-care ability was initially used to explain health-promoting behaviors in patients with chronic diseases, it has been gradually extended and applied to areas such as medication adherence and health behavior control. However, the impact on self-care competencies resulting from physical exercise in older age groups has yet to be explored. In addition, the mediating role of self-care ability on spiritual well-being has yet to be investigated. This study constructs a model of factors influencing spiritual well-being, elucidates the mechanisms by which physical exercise affects spiritual well-being in older adults, and provides new empirical evidence for the critical role of self-care competencies in middle-aged and older adult populations.

This study constructed the mediating role of self-care ability in physical exercise and spiritual well-being in middle-aged and older adults. It is clear from the results that physical exercise significantly affects the spiritual well-being of the elderly. It may be that it enhances the self-care ability of middle-aged and elderly people, makes them feel more self-efficacy, helps them to form scientific health awareness, changes their poor lifestyles, improves their negative emotions, enhances their sense of social value, and reduces the pressure of medical expenses. It also enhances the awareness of physical exercise among the elderly and makes it a part of life to enhance people’s health-promoting behaviors and promote active aging.

### Limitations and future directions

Due to the limitations of this study, these results are suggestive rather than conclusive. First, we extracted the study sample from older adults in one province. Although this has little impact on the study results, it will affect the generalization of the findings. Future studies could expand the sample group to make the results more representative and widespread. Second, this study used a cross-sectional design to collect data and establish the link between physical exercise, self-care ability, and spiritual well-being. Statistical mediation analysis provides a detailed examination of the potential links between the variables being evaluated, while causality is unreliable [67]. Therefore, future studies should use a longitudinal research design to investigate the relationship between variables.

## Conclusion

This study aimed to understand the relationship between physical exercise and spiritual well-being and the mediating role of self-care ability. The results of this study showed that physical exercise has a positive effect on the spiritual well-being and self-care ability of the elderly and influences the spiritual well-being index through the mediating effect of self-care ability. Physical exercise is an active way of activity, which is conducive to the physical and spiritual health and well-being of the elderly under the advocacy of “active aging”. In response to the national advocacy of “active aging”, it is important to find sources of meaning for older adults to improve their sense of meaning to promote successful aging in the future.

## Supporting information

S1 File Data. (SAV)
